# Structure–Function Insights into Immune Receptors Drive Innovation in CAR-T Cell Therapy

**DOI:** 10.3390/cimb48060552

**Published:** 2026-05-24

**Authors:** Tian Xia, Changhe Wei, Xiaofan Chen

**Affiliations:** 1 School of Traditional Chinese Medicine, Jiangxi University of Chinese Medicine, No. 1688, Meiling Avenue, Xinjian District, Nanchang 330004, China; xt21194@163.com; 2 Panxi Crop Improvement Key Laboratory of Sichuan Province, Xichang University, Anning Campus, No. 1 Xuefu Road, Anning Town, Xichang 615013, China; hantanheying@126.com

**Keywords:** chimeric antigen receptor (CAR) T cells, T-cell receptor, signal transduction, adoptive cellular immunotherapy, structural immunology, therapeutic engineering

## Abstract

Chimeric antigen receptor T-cell (CAR-T) therapy has emerged as the most transformative cellular immunotherapy modality, with its evolutionary trajectory intrinsically coupled to advances in immune receptor structure–function paradigms. Recent technological breakthroughs have yielded unprecedented mechanistic insights into immune receptors. Cryo-electron microscopy, single-cell omics, and structural biology have revealed the molecular architecture and functional dynamics of key receptors, including T-cell receptors (TCRs) and B-cell receptors (BCRs). This comprehensive review systematically integrates the latest discoveries in immune receptor structure–function relationships, emphasizing the mechanistic underpinnings of receptor diversity generation, signal transduction networks, and their direct translational impact on CAR-T therapeutic optimization. We critically examine the innovative design principles governing fourth-generation CAR-T cells, delineate breakthrough strategies for overcoming solid tumor immunoresistance, and analyze the synergistic potential of CAR-T and TCR-T technological convergence. Particular attention is devoted to elucidating how fundamental immune receptor research can be harnessed to address the tripartite challenges of safety, efficacy, and persistence that currently constrain CAR-T clinical applications. This review establishes a mechanistic framework for developing next-generation CAR-T technologies grounded in immune receptor biology and provides strategic insights for accelerating cellular immunotherapy clinical translation.

## 1. Introduction

Chimeric antigen receptor T-cell (CAR-T) therapy stands as the flagship of adoptive cellular immunotherapy, achieving unprecedented success in hematologic malignancies through the systematic application of structure–function insights derived from natural immune receptors [1]. As of November 2025, seven second-generation CAR-T products have been approved by the FDA [2], including Aucatzyl approved in November 2024 and six others approved previously. However, further advancement of CAR-T technology, particularly breakthroughs in solid tumor treatment, safety control, and therapeutic durability, relies fundamentally on deeper understanding of immune receptor structure–function relationships and their systematic translation into innovative engineering strategies.

The T-cell receptor (TCR)-CD3 complex serves as the core machinery for T-cell recognition and response, and its structural and functional properties (detailed in Section 2.1) provide crucial insights for CAR engineering [3]. CARs, as artificially designed immune receptors, ingeniously combine the high-specificity recognition capability of antibodies with the potent cytotoxic function of T cells. The evolution from first-generation CARs, which provided a proof-of-concept but lacked sufficient activation signals [4], to fourth-generation T cells that were redirected for universal cytokine-mediated killing (TRUCK) cells with complex engineered designs [5] reflects our deepening understanding of immune receptor functional mechanisms. The evolution from first- to fourth-generation CARs reflects our deepening understanding of immune receptor functional mechanisms, with different costimulatory domain designs (e.g., CD28 vs. 4-1BB) exerting distinct effects on T cell persistence and effector function (detailed in Section 4.2) [6]. Recent mechanistic studies have provided crucial insights into optimizing third-generation CAR design for enhanced persistence. Guedan et al. demonstrated that combining ICOS and 4-1BB intracellular domains in third-generation CARs displayed superior antitumor effects and increased persistence compared to conventional second-generation constructs [7]. ICOS significantly enhanced CD4+ T cell persistence, which subsequently supported CD8+ T cell longevity through helper effects. Importantly, their findings revealed that the membrane-proximal positioning of the ICOS domain was critical for optimal function, establishing structure–activity principles for rational third-generation CAR engineering. Complementing these mechanistic insights, clinical trials evaluating third-generation GD2-specific CAR-T cells in patients with metastatic melanoma and other solid cancers have demonstrated the feasibility and safety of CAR-T therapy in solid tumor settings, with evidence of immune activity and improved CAR-T cell expansion when optimized manufacturing procedures were employed [8]. Together, these mechanistic and clinical findings underscore both the potential and the limitations of third-generation designs [9]. They naturally motivate the development of fifth-generation CARs that integrate additional signaling modules to overcome exhaustion and enhance long-term persistence. Building on these insights, fifth-generation CARs introduce an additional signaling module that directly incorporates elements of cytokine receptor pathways into the CAR backbone [10].

Currently, CAR-T therapy faces critical challenges in expanding from hematologic malignancies to solid tumors. The complex microenvironment of solid tumors, characterized by antigen heterogeneity, immunosuppressive factors, and poor T-cell infiltration, poses higher functional requirements for CAR-T cells [11,12]. Clinical trials of CAR-T therapy in solid tumors have shown limited efficacy, with challenges including on-target/off-tumor toxicity, T-cell exhaustion, and inadequate tumor penetration [13]. Simultaneously, safety concerns such as cytokine release syndrome (CRS) and immune effector cell-associated neurotoxicity syndrome (ICANS), along with issues of long-term CAR-T cell persistence and memory formation, remain significant barriers to widespread clinical application [14,15]. CRS, characterized by elevated levels of inflammatory cytokines including IL-6, IFN-γ, and TNF-α, occurs in 70–90% of CAR-T recipients, with severe cases (grade ≥ 3) occurring in 2–15% of patients depending on the specific product, and this can be life-threatening in severe cases [16]. Addressing these challenges requires a deeper understanding of immune receptor structure–function relationships and translating these insights into innovative CAR design strategies. Recent advances in understanding TCR mechanobiology, including how mechanical forces influence TCR triggering and signaling [17], and the discovery of TCR clustering and microcluster formation [18], provide new perspectives for optimizing CAR architecture. Additionally, insights into T-cell metabolism, epigenetic regulation, and differentiation states are being incorporated into next-generation CAR-T cell manufacturing protocols. This comprehensive review systematically examines the latest advances in immune receptor structure–function research. We particularly focus on analyzing how these fundamental discoveries guide innovative developments in CAR-T technology, providing scientific rationale for the next generation of safer and more effective CAR-T therapeutics. While fourth-generation TRUCKs and logic-gated CARs have entered clinical evaluation, many of the discussed approaches—including mechanosensitive CARs, QRICH1/MICL-based regulatory modules, and fifth-generation JAK/STAT-integrating designs—remain at the preclinical or conceptual stage and lack clinical validation.

## 2. Latest Insights into Immune Receptor Structure and Function and Their Implications for CAR Design

**Figure 1:**
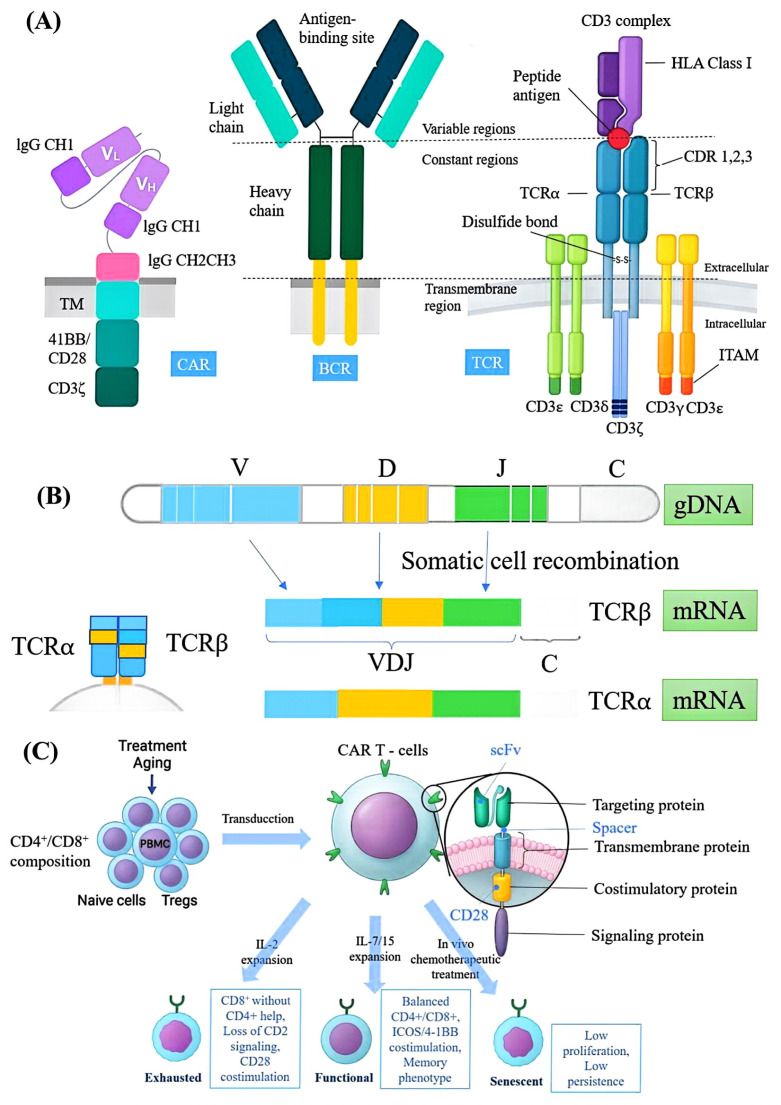
Structural and functional insights into immune receptors inform CAR-T cell engineering. (**A**) Comparison of CAR, B-cell receptor (BCR), and T-cell receptor (TCR) structures, and cellular components of the TCR-CD3 complex. The overview highlights antigen-binding sites, variable/constant regions, and transmembrane domains, while the high-resolution structure shows the αβ TCR heterodimer with CD3 dimers, delineating CDR1-3 and ITAMs. (**B**) Functional insights from receptor diversity: TCRα VJ and TCRβ VDJ recombination generate structural diversity in CDR3 loops. (**C**) Therapeutic CAR-T cell products exhibit inherent functional heterogeneity post-expansion, necessitating precise cytokine conditioning to optimize effector phenotypes. As illustrated by the modular architecture of chimeric antigen receptors (CARs), comprising extracellular scFv domains (derived from antibody V regions), hinge/transmembrane segments, and intracellular signaling domains (CD3ζ ITAMs coupled with costimulatory motifs like CD28 or 4-1BB). Abbreviations: TCR, T cell receptor; CAR, chimeric antigen receptor; ITAM, immunoreceptor tyrosine-based activation motif; CDR, complementarity-determining region; scFv, single-chain variable fragment; and TM, transmembrane domain.

The comprehensive understanding of immune receptor architecture and activation mechanisms has established a robust foundation for rational CAR engineering approaches. These structural and functional insights collectively demonstrate how fundamental immunological principles can be systematically translated into innovative therapeutic designs, as illustrated in the integrated framework presented in Figure 1.

### 2.1. Structural Foundation of the TCR-CD3 Complex and CAR Design Principles

#### 2.1.1. Guidance Significance of High-Resolution Structural Studies for CAR Engineering

The recently resolved cryo-electron microscopy structure of the complete TCR-CD3 complex has provided unprecedented molecular-level guidance for CAR design [19,20]. This structural breakthrough elucidates critical molecular events during TCR activation, establishing a foundational framework for understanding and optimizing CAR signal transduction mechanisms. The high-resolution structure reveals the precise spatial organization of the TCR-CD3 complex [8]. It also shows the conformational changes upon peptide–MHC engagement and the subsequent propagation of activation signals through the CD3 subunits. The structural characteristics of TCR variable regions provide a reference framework for optimizing single-chain variable fragments (scFv) and other antigen-binding domains in CARs. The complementarity-determining region (CDR) loops of the TCR demonstrate how conformational flexibility can be balanced with binding specificity, informing the design of CAR antigen-binding domains with improved affinity and reduced immunogenicity [8]. The optimization of CAR antigen-binding affinity based on TCR-like kinetic parameters is discussed in detail in Section 3.1.2 [21]. Despite these important structural insights, several limitations should be acknowledged. First, the cryo-EM structures represent static snapshots of the TCR-CD3 complex, whereas TCR activation is a highly dynamic process involving conformational fluctuations, lipid bilayer interactions, and force-dependent transitions. These dynamic features are not fully captured by current structural models. Second, the translation of these structural findings into CAR engineering remains largely empirical, with a significant gap between high-resolution molecular details and rational CAR design principles. Third, most structural studies have been conducted in isolation systems (e.g., detergent micelles or synthetic lipid bilayers), which may not fully recapitulate the native membrane environment of T cells. Future integrative approaches combining structural biology with molecular dynamics simulations and functional validation will be necessary to bridge this gap.

The dimerization mechanism of TCR transmembrane domains has inspired stability-enhanced designs for CAR transmembrane regions. Structural analysis reveals that the TCR α and β chain transmembrane domains form stable interactions that are crucial for receptor assembly and surface expression [22]. These insights have led to the development of optimized CAR transmembrane domains that incorporate similar stabilizing features, including the use of CD28 or CD8α transmembrane sequences that promote proper CAR assembly and reduce tonic signaling [23,24]. The spatial arrangement of immunoreceptor tyrosine-based activation motifs (ITAMs) within CD3 subunits provides a template for optimizing CAR intracellular domains. The TCR-CD3 complex contains ten ITAMs distributed across the CD3γ, CD3δ, CD3ε, and CD3ζ chains, with specific spacing and orientation that ensures efficient signal propagation [25]. This architectural principle has informed the design of multi-ITAM CAR constructs and the optimization of ITAM spacing within the CD3ζ signaling domain [26].

Structural studies have revealed that PD-1 functions as a dimer on the T-cell surface through transmembrane domain interactions, providing novel insights for developing self-regulatory CAR systems [27,28]. This discovery has inspired the design of CARs with built-in regulatory mechanisms that can modulate activation strength based on antigen density. Such designs incorporate PD-1-derived transmembrane elements that enable CAR dimerization and enhanced signaling in high-antigen environments (tumor sites) while maintaining relative quiescence in low-antigen settings (normal tissues) [29]. Furthermore, this structural understanding has led to the development of “smart” CARs that incorporate logic gates based on receptor clustering dynamics. These advanced constructs can differentiate between healthy and malignant tissues based on antigen presentation patterns, potentially reducing on-target/off-tumor toxicity while maintaining therapeutic efficacy [30,31]. Logic-gated CAR systems are discussed in detail in Section 3.2. The integration of dimerization-dependent activation mechanisms represents a significant advancement in CAR safety engineering, offering a molecular-level solution to one of the most pressing challenges in CAR-T cell therapy.

#### 2.1.2. Applications of Mechanosensing Mechanisms in CAR Signal Regulation

Mechanobiological studies of the TCR-CD3 complex have revealed multiple activation models that provide crucial insights for sophisticated CAR signal regulation design. The emerging understanding of TCR mechanosensing has demonstrated that T-cell activation is not merely dependent on biochemical recognition but also involves mechanical forces generated during immunological synapse formation [17,32]. Recent studies have demonstrated the critical role of mechanical force in TCR specificity regulation. For instance, research has revealed that natural TCRs exploit mechanical force to form optimal catch bonds with cognate antigens through a mechanically flexible TCR-pMHC binding interface [33]. This interface enables force-enhanced CD8 coreceptor binding through sequential conformational changes. This mechanobiological mechanism has profound implications for CAR engineering, as engineered high-affinity TCRs often create rigid interfaces that prevent force-induced conformational changes, leading to cross-reactivity with self-antigens and off-target toxicity. The clustering model has been successfully applied to CAR optimization through antigen density-sensitive designs that generate enhanced activation signals in high-antigen-density tumor sites while maintaining relative quiescence in normal tissues [34,35]. Furthermore, co-localization enhancement strategies involving structural optimization of CARs to promote receptor clustering have demonstrated improved signal transduction efficiency and therapeutic index [36]. The segregation model has guided CAR design strategies that exploit membrane microdomain separation mechanisms to enhance signal specificity and exclude inhibitory receptors from activation clusters [37].

The mechanosensing principles underlying TCR activation have inspired the proposal of next-generation CARs with force-sensitive capabilities that could potentially preferentially activate under the unique biomechanical conditions of the tumor microenvironment (TME) [38,39]. These proposed mechanically responsive CARs incorporate structural elements that undergo conformational changes in response to mechanical stress, enabling dynamic signal modulation based on the physical properties of target tissues. The integration of catch-bond mechanisms, where receptor-ligand interactions are stabilized by mechanical force, has been suggested for the design of CARs with enhanced discrimination between high-avidity tumor targets and low-avidity normal tissue interactions [40,41]. Additionally, the application of dynamic signal regulation through mechanosensing remains an exploratory area [42,43]. It may eventually lead to “smart” CARs that can modulate their activation state in real-time based on the mechanical environment, potentially reducing off-target effects while maintaining robust antitumor activity.

### 2.2. Guidance from Immune Receptor Diversity Mechanisms for CAR Library Construction

#### 2.2.1. Applications of TCR Diversity Generation Mechanisms in CAR Optimization

The synergistic action of TCR α and β chains in antigen recognition provides crucial insights for sophisticated CAR design strategies, particularly in developing constructs with enhanced tumor specificity and reduced off-target effects [44,45]. The dual-chain recognition principle underlying TCR function has inspired the development of bispecific CAR architectures that incorporate two distinct antigen-binding domains, enabling Boolean logic operations for tumor recognition. These designs leverage the natural cooperativity observed in TCR αβ heterodimers to create CARs that require engagement of multiple tumor-associated antigens for full activation, thereby improving the therapeutic index [30,31]. Variable domain optimization strategies, informed by the structural diversity mechanisms of TCR variable regions, have enabled systematic enhancement of CAR antigen-binding domain affinity and specificity through directed evolution and computational design approaches [30,46]. For example, the application of TCR-derived CDR grafting techniques has led to the development of TCR-like CARs that can recognize intracellular peptide antigens presented on MHC class I molecules, expanding the targetable antigen repertoire beyond surface proteins to include viral antigens and neoantigens [9,47].

CDR engineering strategies, particularly those focused on CDR3 loop optimization, have emerged as powerful tools for refining CAR antigen-binding characteristics. The structural principles governing TCR–peptide–MHC interactions have informed rational design approaches for improving CAR scFv performance [48]. Advanced computational methods, including machine learning algorithms, now enable the rational design of CAR antigen-binding domains with predicted binding affinities (detailed optimization strategies are presented in Section 3.1.2) [49,50]. These computational approaches, combined with high-throughput screening methodologies, have facilitated the systematic optimization of CAR libraries, enabling the identification of lead candidates with superior therapeutic properties while minimizing the risk of autoimmune cross-reactivity [51]. The application of artificial intelligence to predict optimal CAR scFv sequences and binding kinetics represents an emerging frontier in this area. Recent advances in AI applications for biological systems have been reviewed [52].

#### 2.2.2. Insights from Evolutionary Constraint Mechanisms for CAR Safety Design

The evolutionary constraint mechanisms governing immune receptor development provide fundamental principles for engineering CAR safety features that mimic natural tolerance mechanisms while maintaining therapeutic efficacy [53,54]. Self-tolerance mechanisms derived from thymic selection processes offer sophisticated frameworks for predicting and mitigating CAR autoreactivity [55,56]. This can be achieved through computational modeling of T-cell repertoire cross-reactivity patterns. These evolutionary insights have informed the development of advanced safety switch designs that incorporate natural immune tolerance checkpoints, including programmable death switches activated by small molecules and engineered regulatory circuits that respond to physiological danger signals [57,58]. For example, the integration of FOXP3-derived regulatory elements into CAR constructs has enabled the creation of self-limiting therapeutic systems that automatically downregulate CAR expression in response to excessive activation, thereby preventing CRS while preserving antitumor activity [59,60]. Additionally, biomimetic approaches incorporating natural peripheral tolerance mechanisms, such as anergy induction pathways and regulatory T-cell suppressive functions, have been successfully translated into CAR designs with enhanced safety profiles [61].

Balancing selection strategies observed in immune system evolution provides crucial guidance for maintaining functional diversity in CAR libraries while preventing the emergence of dominant clones with limited therapeutic scope [62,63]. The maintenance of functional diversity through polyclonal CAR populations has been shown to enhance therapeutic robustness and prevent tumor immune evasion through complementary recognition mechanisms and differential activation thresholds [64,65]. These diversified CAR designs incorporate redundant recognition pathways and cross-reactive binding domains that collectively maintain therapeutic pressure against heterogeneous tumor populations, effectively recapitulating the natural selection pressures that have shaped immune receptor evolution over millions of years [66,67].

### 2.3. Applications of Emerging Immune Receptor Regulatory Molecules in CAR Function Enhancement

#### 2.3.1. QRICH1 Regulatory Mechanisms for CAR Engineering

QRICH1 (glutamine-rich protein 1) has recently been identified as a novel intracellular checkpoint that negatively regulates CD8+ T cell activation by associating with CARD11 and inhibiting NF-κB signaling following TCR engagement [68]. This regulatory mechanism presents an attractive engineering target for modulating CAR-T activation intensity, functioning as a molecular rheostat that controls antigen-induced proliferation and effector status [69]. The intrinsic checkpoint function of QRICH1 provides a framework for designing safety control systems that dynamically adjust CAR-T responsiveness based on activation context [70]. The development of synthetic gene circuits incorporating QRICH1-based regulatory elements may enable external pharmacological control of CAR-T function, offering clinicians real-time therapeutic modulation capabilities [71]. The therapeutic application of QRICH1-based regulation in CAR-T cells remains entirely preclinical. To date, no CAR-T product incorporating QRICH1-based control has entered clinical trials, and the translational feasibility of this approach requires further validation in relevant in vivo models.

#### 2.3.2. MICL Receptor Mechanisms for CAR-T Inflammatory Regulation

MICL (CLEC12A) is an inhibitory C-type lectin receptor containing immunoreceptor tyrosine-based inhibitory motifs (ITIMs) in its cytoplasmic tail, which recruit the phosphatases SHP-1 and SHP-2 to restrict inflammatory pathway activation [72]. Engineering CAR-T cells to incorporate MICL-derived regulatory modules offers strategic advantages for mitigating CAR-T-associated toxicities, particularly CRS and ICANS [73]. The integration of MICL regulatory circuits enables the development of CAR-T cells with improved inflammatory balance, allowing maintenance of robust antitumor effector functions while simultaneously controlling excessive inflammatory responses that lead to severe toxicity. For example, studies have demonstrated that MICL deficiency results in markedly exacerbated inflammation due to inappropriate activation of myeloid cells, suggesting that engineering MICL-based regulatory modules into CAR-T constructs could provide essential tissue protection functions and reduce treatment-related organ damage [74]. Additionally, MICL’s natural role as an inhibitory receptor for damage-associated molecular patterns, particularly monosodium urate crystals released from damaged cells, positions it as an ideal candidate for developing self-regulating CAR-T platforms that automatically modulate inflammatory output based on cellular damage signals [75].

As with QRICH1, MICL-based regulatory modules have not yet been tested in any CAR-T clinical trial. The proposed benefits for mitigating CRS and ICANS remain speculative until validated in appropriate preclinical models and, ultimately, in human studies. Collectively, these structural and functional insights establish a mechanistic foundation for understanding how natural immune receptors achieve their remarkable specificity and signaling efficiency. Building upon this foundation, the next section examines how these principles have been translated into innovative CAR engineering strategies.

## 3. Structure–Function Insights Drive CAR-T Cell Engineering Innovation

Leveraging the structural and functional insights discussed above, researchers have developed several generations of CAR-T cells with progressively sophisticated designs. The following sections detail these engineering innovations, beginning with fourth-generation CAR-T cells (TRUCKs).

### 3.1. Innovative CAR Structural Design Based on Immune Receptor Mechanisms

#### 3.1.1. Revolutionary Breakthroughs in Fourth-Generation CAR-T Cells

**Figure 2:**
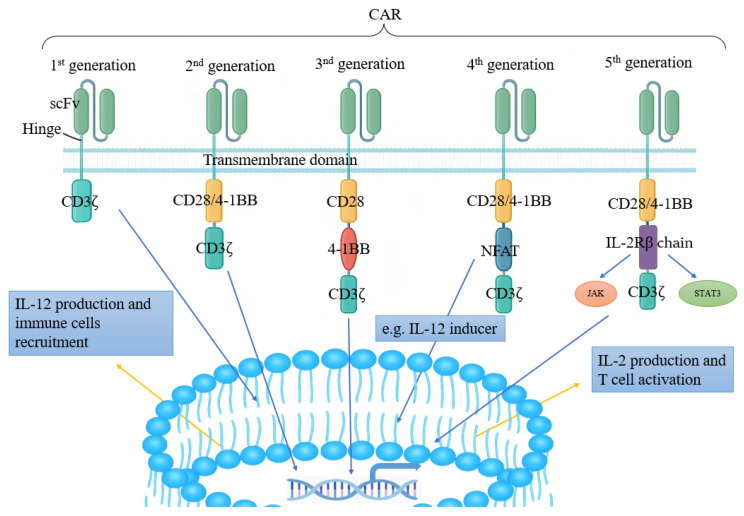
Generational evolution and structural innovation of CAR technology. First-generation CARs: scFv antigen-binding domain linked via transmembrane region to CD3ζ intracellular signaling domain. Second-generation CARs: add one costimulatory domain (e.g., CD28 or 4-1BB) to enhance activation and persistence. Third-generation CARs: incorporate dual costimulatory domains (commonly CD28 plus 4-1BB). Fourth-generation CARs (TRUCKs): based on second-generation design with added transgenic cytokine expression modules (e.g., IL-12) and safety switches. Fifth-generation CARs: structure integrates intracellular signaling via transcription factor (e.g., STAT3) to activate JAK/STAT pathways, enabling autonomous cytokine secretion and enhanced proliferation. Abbreviations: NFAT, nuclear factor of activated T cells; TRUCK, T cells redirected for universal cytokine-mediated killing.

Fourth-generation CAR-T cells, also known as TRUCK cells, represent a paradigm shift in cellular immunotherapy by incorporating sophisticated multi-functional capabilities that address the limitations of earlier CAR generations [76]. These advanced constructs integrate antigen recognition, T-cell activation, cytokine secretion, and immune modulation functions within a single engineered cell platform through the strategic incorporation of inducible transgene expression systems controlled by the nuclear factor of activated T cells (NFAT) responsive elements (Figure 2) [77]. The NFAT-driven design capitalizes on natural TCR downstream signaling pathways to ensure that therapeutic molecule expression occurs exclusively upon tumor antigen engagement, thereby enhancing both safety and therapeutic specificity. For example, T cells engineered with a melanoma-specific TCR and interleukin-18 under NFAT-sensitive promoter control demonstrated antigen-dependent cytokine production that occurred only upon exposure to antigen-positive tumor cells, not antigen-negative cells [78].

The antigen-dependent activation mechanism enables TRUCK cells to function as living drug factories that reshape the TME through localized secretion of immunomodulatory cytokines such as interleukin-12 and interleukin-15, which counteract immunosuppressive conditions and promote endogenous immune cell activation [79]. For example, Li et al. (2021) demonstrated that co-expression of IL-7 and CCR2b significantly enhances CAR-T cell survival and infiltration in solid tumors [80]. This conditional expression system safely modulates the tumor microenvironment. It enhances CD8+ T cell infiltration and prolongs survival without the severe systemic toxicity seen with constitutive cytokine expression. These engineered cells maintained their cytotoxic specificity while exhibiting superior migration abilities and sustained antitumor activity through the synergistic effects of IL-7-mediated survival signals and CCR2b-directed chemotaxis [81]. The revolutionary impact of fourth-generation CAR-T technology extends beyond hematologic malignancies to solid tumor applications (Table 1), where their ability to modulate the hostile TME and promote vascular normalization represents a critical advancement toward overcoming the immunosuppressive barriers that have historically limited CAR-T efficacy in solid malignancies [82].

#### 3.1.2. Refined Engineering and Optimization Strategies for CAR Domain Architecture

The systematic optimization of CAR structural domains translates fundamental immune receptor biology into precision-engineered platforms [87]. Each component requires meticulous calibration to achieve optimal efficacy while maintaining safety. Antigen-binding domain optimization has emerged as a critical determinant of CAR function, where intermediate-affinity designs based on natural TCR–antigen interaction kinetics prevent overstimulation-induced T cell exhaustion while promoting serial target cell engagement [88]. For example, engineering strategies that incorporate modular antigen recognition domains capable of simultaneous multi-epitope binding have demonstrated superior resistance to tumor antigen escape mechanisms, as evidenced by tandem CAR designs targeting both HER2 and IL13Rα2 in glioblastoma patients [87]. These domain-specific advances collectively demonstrate the evolution from empirical CAR design toward mechanistically informed optimization strategies (Table 2). Recent studies have demonstrated that balancing activation and costimulation signals within CAR architectures can fine-tune signaling dynamics to enhance therapeutic potency while maintaining T cell persistence [89]. This approach emphasizes that CAR optimization is not simply about maximizing signal strength, but rather about achieving the appropriate signal quality and kinetics for sustained therapeutic efficacy.

Transmembrane domain engineering has evolved beyond simple structural stability. Strategic selection of transmembrane sequences from different receptor families now enables precise modulation of basal activation levels and membrane localization patterns [105]. Intracellular signaling domain innovation has progressed toward highly modular architectures that permit flexible functional programming through optimized costimulatory domain combinations and ITAM spacing arrangements derived from natural TCR signaling studies [106]. Recent breakthroughs in intracellular domain engineering have demonstrated the transformative potential of incorporating modified CD3 signaling elements into CAR architectures. For example, Xu et al. revealed that integrating a modified CD3ε intracellular domain, termed EB6I, into conventional CAR structures significantly enhances antigen sensitivity and sustains cytotoxic activity by improving immune synapse formation and signal transduction coordination [107]. This innovative approach addresses critical limitations of conventional CARs, including poor antigen sensitivity and suboptimal intracellular signaling molecule coordination, and has shown promising efficacy across various tumor models, including both solid tumors and hematological malignancies [108].

#### 3.1.3. Fifth-Generation CAR-T Cells: Signal-3 Integration

Unlike the fourth-generation TRUCKs that emphasize conditional cytokine delivery to reshape the TME, fifth-generation CARs focus on intrinsically wiring cytokine receptor signaling into the CAR backbone [86]. By fusing a truncated IL-2Rβ cytoplasmic domain between the costimulatory module (such as CD28) and CD3ζ, together with a STAT3/5-recruiting YXXQ motif at the CD3ζ tail, these constructs enable antigen-dependent engagement of the JAK-STAT pathway in parallel with conventional CD3ζ and costimulatory activation. This integrated design equips CAR-T cells with intrinsic “signal 3”, thereby supporting sustained proliferation, enhanced metabolic fitness, and memory-biased differentiation. Preclinical models have demonstrated improved persistence and reduced exhaustion, highlighting the translational promise of these JAK/STAT-integrating architectures for future clinical development [109]. Despite these promising features, fifth-generation CARs also raise important safety concerns that warrant careful consideration. Constitutive or semi-constitutive JAK-STAT signaling may lead to cytokine-independent proliferation, which could theoretically increase the risk of clonal expansion or even malignant transformation over the long term. Furthermore, the addition of cytokine receptor signaling domains may potentiate cytokine release syndrome (CRS) and immune effector cell-associated neurotoxicity syndrome (ICANS) by amplifying inflammatory cytokine production in an antigen-dependent but uncontrolled manner. In addition, the prolonged survival signals provided by JAK-STAT activation may interfere with normal T-cell contraction and memory homeostasis, potentially leading to persistent CAR-T cell accumulation and late toxicities. Therefore, careful preclinical modeling, including long-term safety studies in immunocompetent animal models, and the incorporation of inducible safety switches (e.g., iCaspase9 or truncated EGFR) are essential before clinical translation of these fifth-generation designs.

It should be noted that among the CAR generations discussed, only second-generation CARs have achieved widespread clinical approval and commercial success. Third-generation CARs have shown mixed results in clinical trials with no clear superiority over second-generation designs, while fourth-generation TRUCKs and fifth-generation JAK/STAT-integrating CARs remain primarily in preclinical or early-phase clinical investigation. Mechanosensitive CARs and logic-gated systems, despite their conceptual appeal, face substantial barriers to clinical translation, including manufacturing complexity, regulatory uncertainty, and lack of validated safety data in humans.

### 3.2. Design Principles of Logic Gate CAR Systems

The development of synthetic Notch (synNotch) receptor systems has revolutionized programmable T cell engineering by enabling customizable therapeutic response programs that integrate environmental sensing with conditional gene expression, providing a powerful platform for creating sophisticated cellular circuits with logic gate functionality [110]. AND-gate CAR systems demonstrate enhanced precision targeting through dual-antigen dependence, where T cell activation requires simultaneous recognition of two distinct tumor-associated antigens. This approach has been shown to enhance tumor specificity while reducing on-target/off-tumor toxicity, as demonstrated in studies targeting AML through combined CD33 and CD123 recognition [111]. Clinical applications of AND-gate logic have been successfully demonstrated in glioblastoma targeting, where Dual-RevCAR T cells require simultaneous recognition of both EGFR and GD2 antigens via separate RevCAR modules to achieve full activation, significantly improving tumor specificity while minimizing off-target effects (Saleh et al.) [112].

NOT-gate CAR designs incorporate inhibitory signal integration that prevents activation when normal tissue markers are detected, as demonstrated by dual HER2/HLA-A*02 CAR systems that effectively spare normal tissues while maintaining potent antitumor activity against target-negative tumor cells [113]. This is demonstrated by the SENTI-202 CAR-NK cell platform targeting acute myeloid leukemia, which integrates both OR-gate logic (FLT3 OR CD33 recognition) to maximize tumor coverage and NOT-gate logic (EMCN inhibition) to protect healthy hematopoietic stem cells (Garrison et al.) [114]. A practical implementation of OR-gate logic has been demonstrated in prostate cancer therapy through modular T cell-retargeting systems that simultaneously target both prostate-specific membrane antigen (PSMA) and prostate stem cell antigen (PSCA), enabling effective elimination of tumor cells expressing either antigen individually and preventing the emergence of single-antigen escape variants (Table 3) [115].

While the engineering innovations described above have significantly enhanced CAR-T cell potency, safety remains a critical challenge for clinical translation. The next section addresses how structure–function insights can guide safety engineering.

## 4. Structure–Function Insights Guide CAR Safety Engineering

Beyond enhancing therapeutic efficacy, structure–function insights from immune receptors have proven equally valuable for engineering safer CAR constructs. Understanding the molecular basis of immune receptor activation provides crucial strategies for minimizing adverse effects while maintaining therapeutic potency.

### 4.1. Immune Receptor Signaling Architecture Informs Toxicity Prevention

Understanding the structural basis of immune receptor activation has provided crucial insights for engineering safer CAR constructs that minimize adverse effects while maintaining therapeutic efficacy. The molecular architecture of the natural TCR-CD3 complex reveals how signal strength and duration are precisely controlled through structural constraints, particularly the spatial organization of immunoreceptor tyrosine-based activation motifs (ITAMs) and their sequential phosphorylation patterns [132]. This structural understanding has directly informed CAR signaling domain optimization, where strategic ITAM spacing and costimulatory domain positioning can prevent the excessive activation that contributes to CRS. Structure–function studies of co-inhibitory receptors have elucidated the molecular mechanisms underlying immune tolerance [133]. PD-1 recruits SHP phosphatases via its dual SH2 domains, while CTLA-4 competes with CD28 for B7 ligands. These mechanisms provide blueprints for engineering built-in regulatory circuits. These mechanistic insights have informed multiple CAR engineering strategies: siRNA-mediated downregulation of PD-1 and CTLA-4 has demonstrated enhanced CAR-T cell cytotoxicity and functionality [134], while incorporation of inhibitory receptor endodomains such as CD96 into CAR constructs can attenuate T cell activation to prevent excessive responses [135]. Additionally, CAR-T cells designed to target PD-L1 directly have shown efficacy in suppressing solid tumor growth by disrupting the PD-1/PD-L1 inhibitory axis [136].

### 4.2. Costimulatory Receptor Structure Guides Persistence Optimization

The molecular basis of T cell exhaustion and memory formation has been elucidated through structural studies of costimulatory receptor signaling, providing crucial insights for engineering CAR constructs with enhanced persistence. Structural analyses have revealed that 4-1BB signaling promotes TRAF-mediated NF-κB activation and mitochondrial biogenesis pathways that support oxidative phosphorylation and memory T cell formation, while CD28 signaling favors PI3K/Akt activation through its YxxM binding motif that drives glycolytic metabolism and immediate effector function [137]. These mechanistic insights have directly informed rational CAR engineering strategies, where studies have demonstrated superior persistence of 4-1BB-containing CARs compared to CD28-based designs. For example, in patient-derived metastatic prostate cancer models, 4-1BB-containing PSCA-CARs showed superior T cell persistence and disease control compared to CD28-containing CARs [138]. These CARs also exhibited enhanced selectivity for higher tumor antigen density and a reduced exhaustion phenotype. The molecular basis for this enhanced persistence has been elucidated through studies demonstrating that 4-1BB costimulation uniquely activates noncanonical NF-κB signaling pathways, which promote CAR-T cell survival and reduce pro-apoptotic Bim expression [139]. Advanced third-generation CAR designs that combine 4-1BB with optimized CD28 signaling motifs have further demonstrated enhanced expansion, persistence, and resistance to exhaustion in both monospecific and bispecific configurations, showcasing how structure–function insights can be systematically translated into next-generation therapeutic platforms [140]. These findings collectively demonstrate how fundamental understanding of costimulatory receptor architecture drives innovative CAR engineering strategies with improved clinical performance.

While the engineering strategies described above aim to enhance CAR-T cell safety, advanced CAR systems also introduce new potential risks that warrant careful consideration. Fourth-generation TRUCKs that constitutively or inducibly secrete cytokines carry the risk of uncontrolled cytokine release if the inducible system exhibits leakiness or if CAR-T cells persist beyond intended duration. Fifth-generation CARs with integrated JAK-STAT signaling domains may promote cytokine-independent proliferation, raising theoretical concerns about clonal expansion or malignant transformation. Logic-gated and switchable CAR systems, while offering improved specificity, introduce additional genetic elements that could increase immunogenicity or unpredictably interact with endogenous signaling pathways. For genome-edited CAR-T products, off-target editing remains a concern despite advances in CRISPR/Cas9 fidelity. Long-term persistence, while desirable for sustained antitumor activity, also means that any late toxicity—including secondary malignancies—would be prolonged and potentially irreversible without effective elimination mechanisms. These considerations underscore the importance of incorporating robust safety switches into all advanced CAR platforms before clinical testing.

## 5. Future Directions

High-resolution structural biology techniques continue to advance, including cryo-electron microscopy and single-molecule imaging. These methods promise to unveil unprecedented mechanistic insights into immune receptor dynamics that will further refine CAR engineering strategies. The application of these structural insights extends beyond traditional T cell engineering to encompass innovative therapeutic targets, as exemplified by recent discoveries demonstrating that microglial ITAM-Syk signaling pathways represent promising immunotherapeutic targets for neurodegenerative diseases such as Alzheimer’s disease [141]. These findings illustrate how fundamental immunoreceptor structural knowledge can inform therapeutic strategies across diverse pathological contexts, expanding the translational impact of structure–function research. Combining CAR-T therapy with epigenetic modulators may offer synergistic effects for treating refractory hematologic malignancies [142]. The integration of advanced manufacturing technologies with sophisticated CAR designs represents another critical frontier, with automated platforms now enabling large-scale production of complex logic-gated CAR systems. Emerging in vivo CAR delivery technologies also position the field to systematically engineer therapeutic platforms that recapitulate the regulatory networks governing natural immune responses [143]. Beyond the scientific and engineering challenges discussed above, several practical barriers must be addressed to facilitate clinical translation of next-generation CAR-T platforms. Manufacturing complexity increases substantially with each additional genetic modification, and current good manufacturing practice (GMP) compliance for logic-gated or switchable CAR systems remains challenging and costly. Regulatory pathways for these advanced platforms are not yet clearly defined, particularly for systems incorporating inducible gene expression or multiple transgenic components. Without deliberate efforts to simplify manufacturing and demonstrate cost-effectiveness, the most sophisticated CAR designs may remain inaccessible to the majority of patients who could benefit from them.

Translating the structural and mechanistic insights discussed in this review into clinical practice may require addressing several practical considerations, which we outline here as suggestions for future research. First, the optimization of CAR antigen-binding affinity based on TCR-like kinetic parameters (intermediate affinity with rapid off-rates) may be considered as a priority during the preclinical development of CAR-T products targeting solid tumors, as this design principle has been shown to reduce on-target/off-tumor toxicity while preserving serial killing capacity. Second, the incorporation of mechanosensitive elements or logic-gated recognition systems, while promising, currently requires validation in phase I/II trials to establish safety profiles before broader clinical adoption; early-phase trial designs would benefit from including predefined stopping rules and toxicity monitoring protocols specifically adapted for these novel CAR architectures. Third, the QRICH1 and MICL regulatory pathways remain at an early stage of therapeutic translation, though they are mechanistically well-characterized. Clinicians and researchers could be directed toward developing small-molecule-gated systems based on these pathways over constitutive modifications to enable reversible, dose-dependent CAR-T cell control in patients. Fourth, for fourth-generation TRUCK cells, clinical implementation may benefit from a phased approach beginning with well-tolerated cytokine payloads (e.g., IL-15) before progressing to more potent but potentially toxic candidates (e.g., IL-12), with mandatory correlative studies measuring systemic and local cytokine levels to establish therapeutic windows. Finally, the establishment of multicenter registries tracking real-world outcomes of patients treated with structurally engineered CAR-T products (e.g., logic-gated, mechanosensitive, or TRUCK platforms) would be valuable for generating evidence-based guidelines and identifying patient subgroups most likely to benefit from these next-generation technologies. These clinical translation efforts should proceed in parallel with continued basic research to ensure that engineering innovations are grounded in robust biological principles and deliver tangible improvements in patient outcomes.

## Figures and Tables

**Table 1:** Structural characteristics and functional comparison of different generation CAR-T cells.

CAR Generation	Structural Components	Major Functional Advantages	Representative Products	Ref.
First Generation	scFv + CD3ζ	Proof-of-concept for CAR technology; simple design and construction	Early clinical prototypes; no approved products; mainly used in preclinical studies	[83]
Second Generation	scFv + CD28 or 4-1BB + CD3ζ	Increased persistence; higher proliferation capacity	Kymriah, Yescarta, Tecartus, Breyanzi, Abecma, Carvykti, Aucatzyl	[84]
Third Generation	scFv + dual costimulatory domains + CD3ζ	Enhanced effector functions; greater proliferative capacity	KTE-X19 variants; multi-clinical trial candidates; platform-specific developments	[85]
Fourth Generation (TRUCK)	scFv + costimulatory domain + CD3ζ + NFAT-driven transgene	Tumor microenvironment remodeling; enhanced recruitment of endogenous immune cells	Clinical trial candidates: IL-12 TRUCK, IL-15 TRUCK, IL-18 TRUCK; investigational products in Phase I/II trials	[5]
Fifth (JAK/STAT-integrating)	scFv + costimulatory domain + CD3ζ + NFAT-driven transgene	Aims to boost in vivo persistence	CD19-28ζ-IL-2Rβ(YXXQ) designs	[86]

CAR, chimeric antigen receptor; scFv, single-chain variable fragment; TRUCK, T cells redirected for universal cytokine-mediated killing; NFAT, nuclear factor of activated T cells; clinical trial status information for investigational products has been verified against ClinicalTrials.gov as of November 2025. Table 1 summarizes the structural and functional characteristics of different generations of CAR-T cells. It should be noted that all FDA-approved CAR-T products to date belong to second-generation CAR designs, while higher-generation CAR-T cells remain at the preclinical or early-phase clinical investigation stage.

**Table 2:** Structure–function-guided CAR engineering innovations across diverse tumor contexts and their mechanistic foundations.

Tumor Context	Immune Receptor Structural Insights	CAR Engineering Innovation	Mechanistic Rationale	Translational Impact	Ref.
B-cell Hematological Malignancies	TCR αβ heterodimer cooperative binding mechanism; CD3ζ ITAM spatial organization	Optimized CD19/CD22 dual-targeting CARs with sequential ITAM activation	Mimics physiological TCR signal amplification through coordinated ITAM phosphorylation cascades	FDA-approved therapeutics with established clinical efficacy	[90,91]
Multi-Myeloma	BCMA natural ligand (APRIL/BAFF) binding stoichiometry; TCR mechanosensing force sensitivity	High-affinity BCMA CARs with mechanically tuned hinge domains	Force-dependent conformational switches optimize signal transduction under bone marrow mechanical constraints	Clinical breakthrough in plasma cell malignancies	[92,93,94]
Glioblastoma	Blood–brain barrier TCR trafficking mechanisms; CNS-specific checkpoint receptor expression	EGFRvIII CARs with integrated checkpoint inhibition modules	Exploits natural T-cell CNS migration pathways while counteracting local immunosuppression	Phase I/II trials demonstrating CNS bioavailability	[95,96,97]
Solid Tumor	Mechanoreceptor force-sensing domains; integrin-mediated adhesion complexes	Mechanically responsive CARs with force-activated signaling modules	Preferential activation under tumor mechanical stress conditions vs. normal tissue compliance	Preclinical validation of mechanosensitive platforms	[98,99,100]
Immunosuppressive Microenvironments	Co-inhibitory receptor structural architecture; checkpoint signaling pathway organization	Armored CARs with integrated cytokine production and checkpoint disruption	Combines CAR activation with local immunomodulation based on natural T-cell activation requirements	Promising preclinical efficacy in cold tumors	[101,102,103,104]

CAR, chimeric antigen receptor; TCR, T-cell receptor; ITAM, immunoreceptor tyrosine-based activation motif; BCMA, B-cell maturation antigen; APRIL, a proliferation-inducing ligand; BAFF, B-cell activating factor; EGFRvIII, epidermal growth factor receptor variant III; CNS, central nervous system; FDA, Food and Drug Administration.

**Table 3:** Technical characteristics comparison of emerging CAR-T technology platforms.

Technology Platform	Technical Principle	Main Advantages	Application Scenarios	Development Stage	Ref.
Logic-gated CAR	AND/OR/NOT logic gates	Enhanced tumor specificity; reduced on-target/off-tumor toxicity; improved safety profile	Solid tumors with heterogeneous antigen expression; organs with shared antigens	Phase II/III trials	[116,117]
Switchable CAR	Small-molecule-controlled on/off switch	Controllable ON/OFF function; dose-dependent activation; safety switch capability	High-risk solid tumors; dose-escalation studies; safety-critical applications	Phase I/II trials	[118,119,120]
Universal CAR (UniCAR)	Universal tag + bispecific adapter	Off-the-shelf availability; rapid target switching; reduced manufacturing costs	Multitumor types; personalized combination therapy; emergency applications	Phase II trials	[121,122,123]
Armored CAR	Engineered to secrete immunomodulators	Enhanced persistence; improved TME penetration; resistance to immunosuppression	Immunologically cold tumors; TME with regulatory barriers; combination immunotherapy	Phase II/III trials	[124,125,126]
CAR-NK cells	CAR-engineered NK cells	Reduced GvHD risk; off-the-shelf potential; multi-killing mechanisms	Allogeneic applications; elderly patients; bridge therapy	Commercial development	[127,128]
In vivo CAR	In vivo gene delivery	Eliminates ex vivo manufacturing; reduced costs; broader accessibility	Resource-limited settings; rapid deployment; pediatric applications	Preclinical studies	[129,130,131]

TME: Tumor microenvironment; GvHD: Graft-versus-host disease; NK: Natural killer.

## Data Availability

No new data were created or analyzed in this study. Data sharing is not applicable to this article.

## Abbreviations

The following abbreviations are used in this manuscript:

CAR	Chimeric antigen receptor
TCR	T-cell receptor
BCR	B-cell receptor
scFv	Single-chain variable fragment
ITAM	Immunoreceptor tyrosine-based activation motif
CDR	Complementarity-determining region
TM	Transmembrane domain
TRUCK	T cells redirected for universal cytokine-mediated killing
NFAT	Nuclear factor of activated T cells
FDA	Food and Drug Administration
CRS	Cytokine release syndrome
ICANS	Immune effector cell-associated neurotoxicity syndrome
TME	Tumor microenvironment
CNS	Central nervous system
GvHD	Graft-versus-host disease
CR	Complete response
ORR	Overall response rate
BCMA	B-cell maturation antigen
APRIL	A proliferation-inducing ligand
BAFF	B-cell activating factor
EGFRvIII	Epidermal growth factor receptor variant III
MHC	Major histocompatibility complex
HLA	Human leukocyte antigen
NK	Natural killer
QRICH1	Glutamine-rich protein 1
MICL	Myeloid inhibitory C-type lectin-like receptor
ITIM	Immunoreceptor tyrosine-based inhibitory motif
SHP	Src homology region 2 domain-containing phosphatase
PI3K	Phosphoinositide 3-kinase
STAT	Signal transducer and activator of transcription
JAK	Janus kinase
NF-κB	Nuclear factor kappa-light-chain-enhancer of activated B cells
synNotch	Synthetic Notch
PSMA	Prostate-specific membrane antigen
PSCA	Prostate stem cell antigen
TRUCKs	T cells Redirected for Universal Cytokine-mediated Killing
ICOS	Inducible T-cell costimulator
PD-1	Programmed cell death protein 1
PD-L1	Programmed death-ligand 1
CTLA-4	Cytotoxic T-lymphocyte-associated protein 4
TRAF	TNF receptor-associated factor
Tscm	Stem cell-like memory T cells
Tcm	Central memory T cells
